# Cationic CNC-stabilized Pickering emulsions of linseed oil for hydrophobic coatings†

**DOI:** 10.1039/d3ra01412f

**Published:** 2023-06-05

**Authors:** Esther E. Jaekel, Svitlana Filonenko

**Affiliations:** a Max-Planck-Institut fur Kolloid und Grenzflachenforschung, Colloid Chemistry Am Mühlenberg 1 14476 Potsdam Germany esther.jaekel@mpikg.mpg.de +49 331 567 9559

## Abstract

Coating paper with polymers is an efficient way of awarding it with gas-tightness and moisture protection in food packaging applications, but it impedes the recyclability of the both paper and polymer. Cellulose nanocrystals were found to be excellent gas barrier materials, but their hydrophilicity forbids their straightforward application as protective coatings. To introduce hydrophobicity into a CNC coating, this work exploited the capability of cationic CNCs isolated in a one-step treatment with a eutectic medium to stabilize Pickering emulsions in order to include a natural drying oil in a dense layer of CNCs. This way, a hydrophobic coating with improved water vapor barrier properties was achieved.

## Abstract 2

Pickering emulsions of linseed oil stabilized by an cationic cellulose nanocrystals can serve as an environmentally friendly hydrophobic and moisture-protective paper coating.

## Graphical abstract

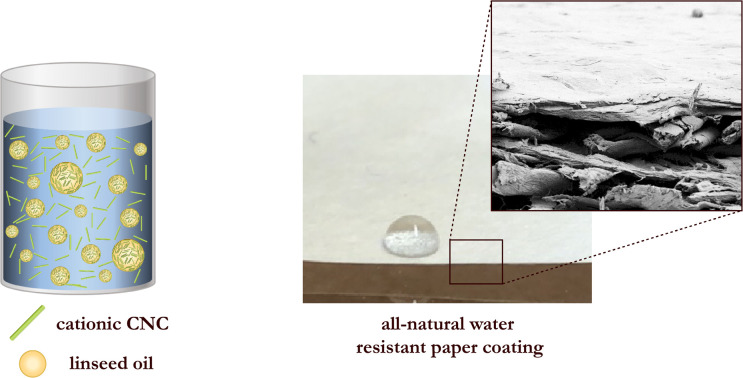

## 1 Introduction

Cellulose nanocrystals (CNCs) are known for their excellent gas barrier properties in nanocomposites as well as self-standing films, owing to their high degree of crystallinity.^1,2^ As a biodegradable and biocompatible barrier material, CNCs hold great potential for sustainable food-packaging solutions. Protective food packaging requires not only low gas (particularly oxygen) permeability, but also resistance against water and humidity. CNC films, however, suffer from hydrophilicity and brittleness, which impedes their straightforward use as packaging films. While they are too brittle to be applied as stand-alone films, CNCs are promising as alternative coatings for paper, which has experienced a revival in the past years as a packaging material to combat plastic pollution. To achieve gas-tightness and moisture resistance, paper is often coated with thin coatings of synthetic polymers, and so recyclability of both paper and plastics are lost.

Various biopolymers have emerged as biodegradable alternative packaging, among them cellulose,^3^ protein,^4^ alginate,^5^ or chitosan^6^ films. While they exhibit good mechanical properties, they all suffer from a high affinity to water that limits their barrier properties. In line with the aim for biodegradability, vegetable oils or natural waxes^7^ have been considered to solve this issue. They pose sustainable alternative hydrophobization to methods like silanizations or coatings with fluorinated synthetic polymers like polyvinylidenefluoride. A positive effect on the water vapor barrier properties through incorporation of vegetable oils into biopolymers has been reported in various studies, often in the context of edible films. The two approaches of combining hydrophilic polymers with hydrophobic oils and waxes are (1) lamination and (2) emulsification. Waxes and oils are difficult to apply in a thin layer due to their solid state or high viscosity, respectively. A coating that is competitive with conventional polymer coatings should be compatible with established processes, requiring a single easily applicable coating. Emulsions facilitate the incorporation of smaller amounts of oil or wax and are easier to process and adjust to established coating processes, in which rheology is a crucial factor. As a drawback, emulsification often requires surfactants,^8^ which can be harmful to human and environmental health. Emulsions stabilized by small particles (Pickering emulsions) are considered more environmentally friendly than surfactant-based emulsions. Some biopolymers, such as proteins, can act as Pickering stabilizers themselves, enabling two-component systems in which the polymer is both film-forming compound and emulsion-stabilizer. For instance, Galus emulsified rapeseed oil (3%) in a soy protein solution, which lead to a decrease of the water vapor permeability of the dried film of up to 20%.^9^ So called drying oils such as tung oil or linseed oil in particular are suited for hydrophobic coatings, since they can be conveniently applied in liquid state and solidify through spontaneous oxidative crosslinking of the polyunsaturated fatty acids that are present in high amounts, forming a hard and water-insoluble film. Submicron coatings of linseed oil, which were achieved by partially washing away the macroscopic coating with hexane, on alginate-based films were 25% less permeable to water than pure alginate films.^10^

In combining their capability to form Pickering emulsions with their excellent barrier properties, CNCs can fulfill a double role of emulsifying hydrophobizing agents while simultaneously sealing the pores of the coated paper. CNC-stabilized emulsions of natural oils can therefore act as a two-in-one coating to provide gas-tightness and hydrophobicity. In fact, a water vapor barrier paper coating consisting of CNC-stabilized epoxy soybean oil acrylate was successfully fabricated and applied by Liu *et al.*,^11^ who achieved an increased contact angle of 74° and improved water barrier transmission rate compared to coatings with only CNCs. Although based on natural resources, soybean oil, its epoxidation and acrylation involves dangerous and harmful chemicals such as meta-chloroperoxybenzoic acid and acrylic acid. The use of unmodified natural oils would greatly enhance the greenness of the coating. Moreover, the authors used CNCs obtained by homogenization of microcrystalline cellulose which suffer from low colloidal stability due to the absence of a surface charge. The most commonly used CNCs are extracted *via* sulfuric acid hydrolysis and bear a high negative surface charge which prevents their assembly at the oil–water interface due to electrostatic repulsion, so that they can only be used as Pickering stabilizers when their charge is screened by salt addition.^12^ While CNCs obtained *via* HCl hydrolysis can stabilize Pickering emulsions without such constraints,^13^ they also bear no ionic functionalities and therefore tend to agglomerate, complicating their application. In the present work, we use our novel cationic CNCs extracted with a reactive eutectic medium in a one-step process, as reported previously.^14^ In our recent study, we found low gas permeabilities of around 0.9 Barrer for glucose-plasticized CNC films.^15^ Their cationic surface charge ensures a good dispersibility of the CNCs, hence enabling a straightforward application. Compared to sulfate-CNCs, their charge is low enough to be suitable for the application as Pickering stabilizer, as it prevents strong repulsion between the CNCs that would hinder their assembly at the interface. Moreover, the negative net charge found at the interface of triglyceride droplets and water (due to fatty acid impurities^16^ or rearrangements of polar groups to the interface^17^) favors the interfacial adsorption of cationic CNCs over anionic or neutral ones. Here, we use linseed oil-in-water emulsions stabilized by cationically modified CNCs as a protective coating on paper to enhance its water repellency and gas impermeability.

## 2 Results and discussion

Emulsions of 2% linseed oil in water with an excess of CNCs in the aqueous phase were first fabricated using a high-speed lab homogenizer. Emulsion coatings were produced at different CNC : oil ratios and concentrations. CNC contents of 4 mg mL^−1^ and 12 mg mL^−1^, resulting in CNC : oil dry mass ratios of 1 : 4.5 (CL1) and 1 : 1.5 (CL2), were tested in preliminary experiments. Since the oil in the coating may increase the air permeability of the coating due to its low polarity, lower oil ratios of 40% (CL3) and 10% (CL4) of the dry mass (0.5% and 0.6% in the emulsion) were tested afterwards. Here, the effect of the area density (dry mass per unit area of the paper) of the coating on its water barrier properties was investigated. The compositions of all tested samples are summarized in Table 1. With all compositions, stable emulsions are formed that do not phase-separate during the observation period of three months. The high CNC content of CL2 leads to a high viscosity, which facilitates the trapping of air bubbles in the emulsion during the homogenization. CL2 therefore had to be degassed in an ultrasound bath before application. To control the final coating mass and in lack of professional coating equipment, the coating was applied by casting a defined amount onto a pre-wetted filter paper placed in a Petri dish of the same size. Pre-wetting ensures adhesion to the glass and a flat surface that is necessary for a homogeneous distribution of the coating.

**Table 1:** Compositions of the emulsion coatings fabricated

	*ρ*(CNC) mg mL^−1^	Oil in emulsion [vol%]	Dry mass CNC [%]	Dry mass oil [%]
CL1	4	2	18	82
CL2	12	2	40	60
CL3	2.4	0.5	60	40
CL4	5	0.6	90	10

### 2.1 Preliminary coating tests with high-oil content emulsions

**Fig. 1:**
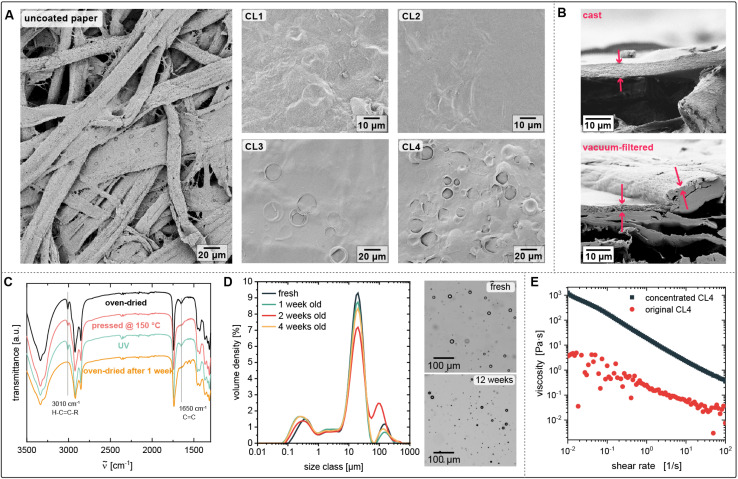
(A) The comparison of SEM images of the surfaces of uncoated paper and the same paper coated with CL1–4 shows complete coverage of pores at the lowest tested density of 0.8 mg cm^−2^ (CL3–4). (B) The cross section of the paper coated by emulsion casting (here of CL3) creates a non-penetrative layer on top of the paper pores, while the vacuum-filtered emulsion coating adapts to the surface of the paper. (C) FTIR spectra of coated paper treated at different conditions. (D) Size distribution curves and optical microscopy images of fresh and aged emulsions. (E) Flow curves of CL4 as used for the presented paper coating and as a concentrated version with the same ratio of CNC/oil.

CL1 and CL2 were applied to filter paper to yield a dry coating mass (CNC and oil) of approximately 1.3 mg cm^−2^ (CL1) or 1.6 mg cm^−2^ (CL2). The paper was dried at 80 °C. A second coated paper sample was pressed in a hot press for 2 h at 150 °C in order to facilitate cross-linking of the linseed oil and flatten the paper. A third sample was subjected to ultraviolet (UV) radiation. Linseed oil spontaneously cross-links in air within approximately one week, becoming dry to the touch. The cross-linking can be observed in the FTIR spectra by following the bands attributed to double bond vibrations. The two main bands that indicate the curing of linseed oil are at 3010 cm^−1^ (*cis ν*(C–H) in C=C–H band) and at 1652 cm^−1^ (–CH=CH– (possibly only *cis*) vibration). The decrease of these bands, see Fig. 1 C, indicates the disappearance of double bonds due to their oxidation or isomerisation from *cis* to *trans*.^18^ Isomerisation activity is regarded a measure for cross-linking, as the process involves the formation of a radical (followed by the addition and elimination of hydrogen), which can be interrupted by the reaction with oxygen. The disappearance of those bands confirms that the linseed oil in the coating is cured within a week after application. While UV irradiation had no observable effect, the heat applied in the hot press facilitated the cross-linking, as observable in the decreasing *cis* C=C double bonds bands. The high temperature, however, caused a strong yellow discoloration of the paper, which was not observed for the RT-dried coatings. The hot-pressing of subsequent samples presented in this study was therefore conducted at a lower temperature and a shorter time, merely aiming to produce a flat surface. Both CL1 and CL2 produced a good coverage of the paper pores (see Fig. 1A). The coatings are approximately 10 μm thick as measured from the cross-sections visualized with SEM. A simple preliminary test in which a water droplet is placed on top of the CL1-coated paper demonstrates the coating's impermeability towards water: The droplet remains on the surface without being absorbed until it evaporates, proving successfully sealed pores and water resistance of the coating. To quantify the hydrophobicity of the coating, the water contact angle (*θ*_w_) was measured in a sessile drop experiment. Uncoated filter paper absorbs water within less than a second (see Fig. S1†), reducing the contact angle to 0°. Coating with linseed oil prolongs this time to 14 s, but cannot prevent the penetration, demonstrating the importance of sealing the paper pores. A paper coated with only CNCs has a *θ*_w_ of 60°. For CL1-coated paper, *θ*_w_ is significantly increased to 99°, qualifying it as a hydrophobic surface. The hot-pressed CL1-coating results in a slightly lower, but still hydrophobic, *θ*_w_ (94°) (Table 2). The reason could be that the surface roughness that allows entrapping of little air cushions is smoothed by the press.

**Table 2:** Water contact angles *θ*_w_ for a pure CNC coating, CL1, and CL1 pressed at 150 °C with a pressure of 2 metric tons

Coating	Water contact angle [°]	
CNC	60.71 ± 0.11	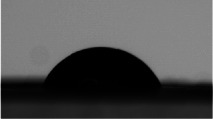
CL1	99.30 ± 2.58	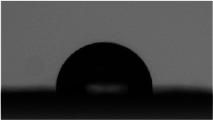
CL1, hot-pressed	94.33 ± 0.57	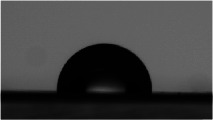

### 2.2 Characterization of lower-oil content coatings

As a high content of nonpolar oil can decrease the coating's resistance against nonpolar permeates such as oxygen, which can, for instance, lead to the spoilage of food, it is of interest to find the lower boundaries of oil content while still providing water resistance. The emulsions with lower oil content CL3 and CL4 and the resulting coatings of varied thickness were investigated more closely.

#### 2.2.1 Emulsion rheology

Homogeneous spreading of the small masses on the paper(for the aspired area densities of 0.8 mg cm^−2^, 1.6 mg cm^−2^ and 2.4 mg cm^−2^) required more diluted emulsions (0.5% oil in water) in this experimental series without specialized coating equipment. For realistic coating applications, however, higher concentrations could be used for instance in blade-casting, which benefits from the shear-thinning properties of the CNCs. Blade-casting requires high viscosities of 1000 mPa s to 10 000 mPa s for a reliable film formation.^19^ Shear-thinning properties of the coating are beneficial as the coating is easily spreadable on the surface when sheared, but stops flowing after being deposited. Fig. 1E shows the flow curves of the diluted CL4 (5.2 mg mL^−1^ CNC, 0.6 mg mL^−1^ oil) and a more concentrated version of the emulsion with the same CNC/oil ratio (12.6 mg mL^−1^ CNC, 1.4 mg mL^−1^ oil). Both emulsions exhibit shear-thinning due to the rod-shaped CNCs in the continuous phase. The diluted emulsion CL4 has a low viscosity over the entire range of shear rates *γ̇*. The more concentrated version reaches a viscosity of 1000 Pa s at low *γ̇*, which is in the required range for blade-casting, and it decreases to 1 Pa s at high *γ̇*, facilitating the spreading. Thus, it is suitable for industrially applied coating procedures.

#### 2.2.2 Emulsion stability

For practical application, a long emulsion stability (in other words, a long shelf life) is important. The stability was investigated exemplary for CL4. Optical microscopy shows emulsion droplets between 1 μm and 20 μm, which are stable for more than 12 weeks (see Fig. 1D). Neither changes of droplet size (through Ostwald ripening or coalescence) or phase separation are observed in this period (Fig. S2†). Stability of CL4 is also confirmed by comparison of the droplet size distribution curves obtained *via* static light scattering. The distribution does not change significantly within the observation period of 4 weeks, with the median diameter *D*_50_ ≈ 16 μm.

#### 2.2.3 Surface properties of the coatings

CL3 and CL4 were tested in terms of their wettability. CL3 and CL4 yield significantly lower contact angles than measured for CL1 (compare Table 3). CL4, containing the lowest dry mass ratio of oil of 10%, displays a *θ*_w_ of 77° ± 4° for the lower area density and 84° ± 12° for the higher area density. Although this does not categorize CL4 as a hydrophobic coating, *θ*_w_ is significantly increased compared to the pure CNC coating. A large deviation of the measured values indicates that the oil is inhomogeneously distributed. CL3, with 40% dry mass oil content, produces a hydrophobic surface with a *θ*_w_ up to 94° for the lowest coating density, which is close to the value reached for the 82% oil coating. The thicker coating of CL3 produces a less hydrophobic coating with a *θ*_w_ of only 87°.

**Table 3:** Contact angles measured for the coatings CL3 and CL4 at different area densities

	Contact angle	Composition	Area density
CL3	94° ± 2°	60% CNC, 40% oil	0.8 mg cm^−2^
CL3	87° ± 2°		2.4 mg cm^−2^
CL4	77° ± 4°	90% CNC, 10% oil	0.8 mg cm^−2^
CL4	84° ± 12°		2.4 mg cm^−2^

**Fig. 2:**
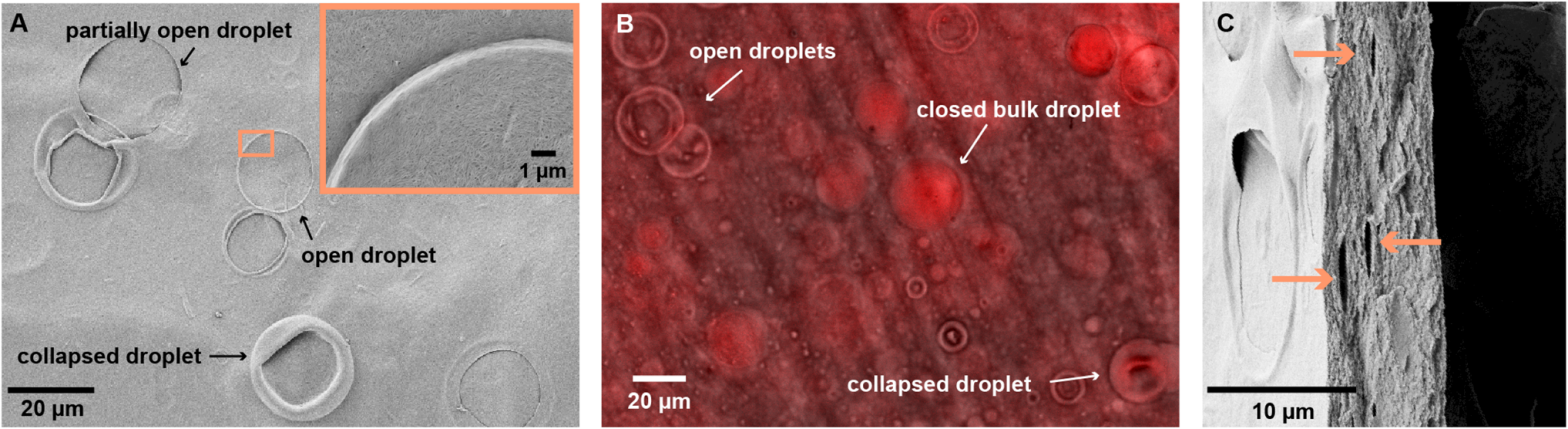
(A) SEM picture of the surface of a CL4-coated paper showing different states of droplets observed on the surface. (B) Confocal microscopy image of a freestanding film of CL3, fabricated with Nile red-stained linseed oil, taken shortly after preparation reveals that surface droplets are depleted of oil, while bulk droplets keep the oil encapsulated during drying. (C) Cross-section of the CL4-coated paper shows elongated, lentil-shaped pores that arise from the oil droplets encapsulated in the bulk of the coating (indicated by orange arrows).

SEM images of the coated surface reveals bubbles on the surface of the coatings, predominantly in CL3 and Cl4. CL1 and CL2, which with their higher oil content had a much higher concentration of oil droplets, barely exhibit bubbles on the surface. Presumably, surface droplets burst and produced a continuous oil film on the surface of the CL1 and CL2 coating, whereas in the more diluted emulsions CL3 and CL4, droplets on the surface occur more sporadically. Here, the surface is characterized by traces of oil droplets that are partially enclosed by CNCs. The amount of open bubbles drastically decreases for higher area densities, which can be explained by the thickness of the coating, as observed in the SEM image of the cross-section: For the lowest coverage of 0.8 mg cm^−2^, the coating thicknesses are 6.9 μm for CL3 and 5.1 μm for CL4, which is lower than the diameter of the biggest droplets in the emulsion of up to 20 μm. Therefore, the droplets which are larger than the cross-section of the film will be exposed to the surface of the coating. As the interfacial tension between the unpolar oil and air is low, the hydrophilic CNCs migrate to the drying edge, as shown in Fig. 2A, and the droplet opens. Presuming that the oil spills across the surface upon opening of the droplet, the higher amount of opened droplets in thinner coatings would explain the higher *θ*_w_ of the thin CL3 coating compared to the thick one.

To investigate this hypothesis, a transparent self-standing film of CL3 was made by casting the emulsion into a Teflon tray and drying it at 80 °C to ensure quick evaporation of the water without starting the cross-linking on linseed oil. The oil phase of the emulsion was stained with Nile red, a lipophilic fluorescent dye, in order to trace its location using confocal laser scanning microscopy (CLSM). The micrographs, which are taken immediately after the film dried, confirm the presence of collapsed surface droplets. This shows that the emulsion droplet is quickly depleted of oil unrelated to its cross-linking, which occurs on a longer time frame. The CLSM image of the film surface, Fig. 2B, reveals fluorescence of Nile red in the oil phase on the entire surface of the film. Oil droplets that are enclosed in the bulk show increased fluorescence intensity, demonstrating an enrichment of oil in the intact droplet. The preservation of bulk droplets is also observed in the SEM images of the cross-section, which reveal lentil-shaped pores (Fig. 2C). In contrast, the inside of the open surface droplets do not exhibit a fluorescence intensity compared to their environment, confirming the depletion of oil. The fluorescence across the whole surface of the film allow the conclusion that the oil is released from the droplet and subsequently spreads across the surface, resulting in the observed hydrophobicity. For lower oil contents, as in CL4, the concentration of droplets on the surface might not be sufficiently high to result in a full surface coverage. The large deviations in *θ*_w_ as observed for the thick coating of CL4 are the consequence.

The cross-section SEM images also show that the coatings form a distinct layer on top of the paper without penetrating and filling the pores between the randomly assembled cellulose fibers. On the one hand, the consequence is a less rough surface than what would be expected from a coating that adapts to the surface morphology of the paper, which decreases the wettability and contact angle. On the other hand, the flexibility and the surface adhesion of the film are low, leading to ruptures observed in SEM images. Adhesion and adaptability to deformation of the paper can be approved by applying the coating *via* vacuum filtration. As shown in Fig. 1B, the coating is sucked into the paper pores at the surface and therefore adapts to the paper morphology.

#### 2.2.4 Water barrier properties of the coated paper

**Fig. 3:**
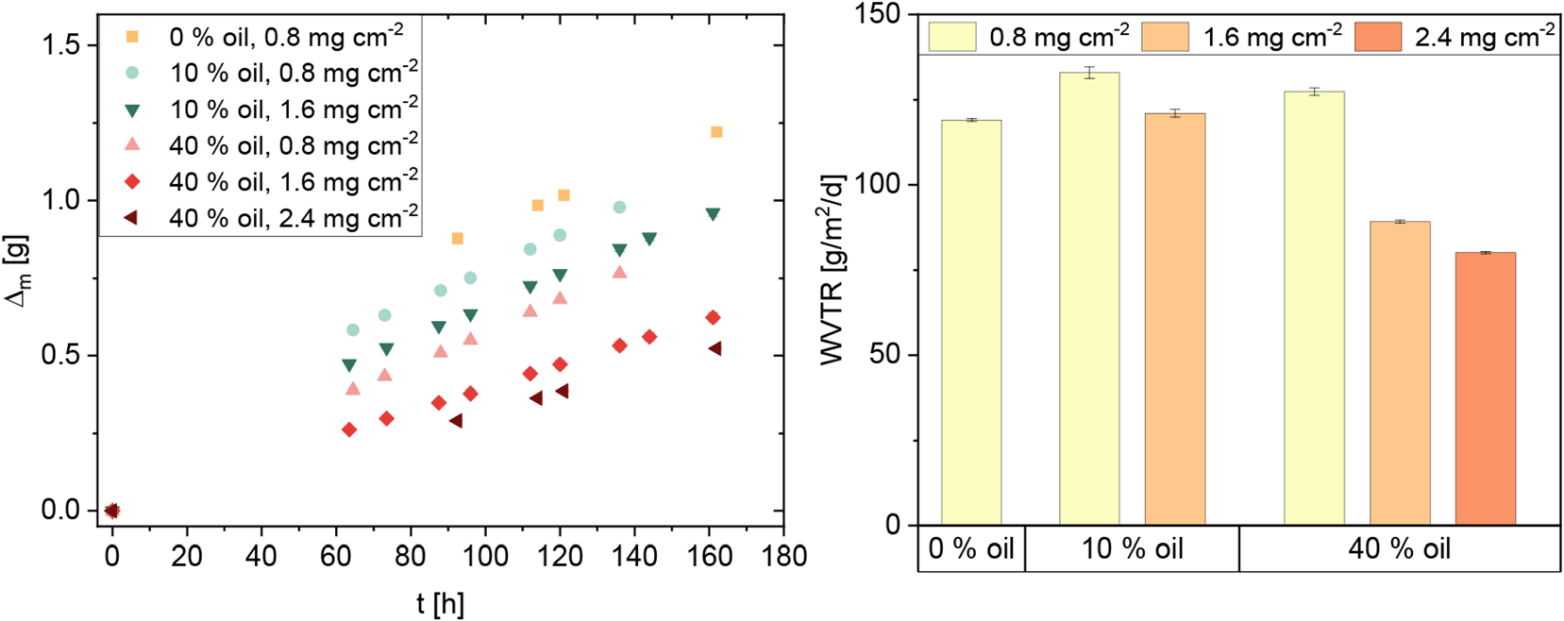
Water gain curves (left) and water vapor transmission rates (WVTR, right) through a filter paper coated with CNC-based coatings with different oil contents and different area densities.

Water vapor permeability of the coatings CL3 and CL4 was quantified by measuring the water vapor transmission rate (WVTR) according to a standardized protocol (German industry norm DIN 53122-1). Here, the water vapor transmission through the coated paper is quantified by recording the mass gain of a desiccant in a chamber that is covered and sealed with the paper. The WVTR can be determined from the slope of the water gain *versus* time curves once a linear regime is reached. The samples were tested in a desiccator filled with a saturated KCl solution, resulting in a climate of 85% relative humidity at 20 °C. The linseed oil-containing coatings CL3 and CL4 are compared to a pure CNC coating (area density: 0.8 mg cm^−2^). The mass gains in the desiccant for the different coatings are shown in Fig. 3 (left). The pure CNC coating (denoted as 0% oil) exhibits the highest water uptake. The total water uptake decreases as the amount of emulsified oil and the area density increase. The WVTR of the pure CNC coating, however, is even lower than the values obtained for both CL3 and CL4 with the same low area density (see Fig. 3(b)). Therefore, the main effect of the oil is an inhibition of the initial high water uptake at the start of the measurement, before the system reaches a constant WVTR. Only for the higher coating thicknesses, a significant effect of the oil content on the WVTR is observed: At medium thickness, the WVTR is decreased from 121 g m^−2^ d^−1^ using CL3 with 10% oil to 89 g m^−2^ d^−1^ with 40% oil. For the thickest coating, the trend persists, with a WVTR of CL4 of 80 g m^−2^ d^−1^*versus* 106 g m^−2^ d^−1^ in CL3. This proves the reduction of WVTR in CNC films through incorporation of linseed oil. The lowest WVTR value reached in this experiment is still high compared to common packaging materials such as polypropylen with a WVTR of 2.5 g m^−2^ d^−1^.^20^ However, the paper used here is a thin filter paper that could be replaced with, for instance, a smoothed and denser packaging paper to improve the WVTR. The inclusion of more oil might further improve the water vapor barrier properties. In combination with future gas barrier testing of the emulsion coatings, a middle ground must be found on which gas barrier and water vapor properties are both in a suitable range. Furthermore, the coating can easily be tuned by dissolving less hydrophilic compounds, such as other biopolymers, in the aqueous phase. For food-related applications, future testing will also include the evaluation of the coatings in terms of grease resistance.

#### 2.2.5 Thermal analysis of the coated paper

**Fig. 4:**
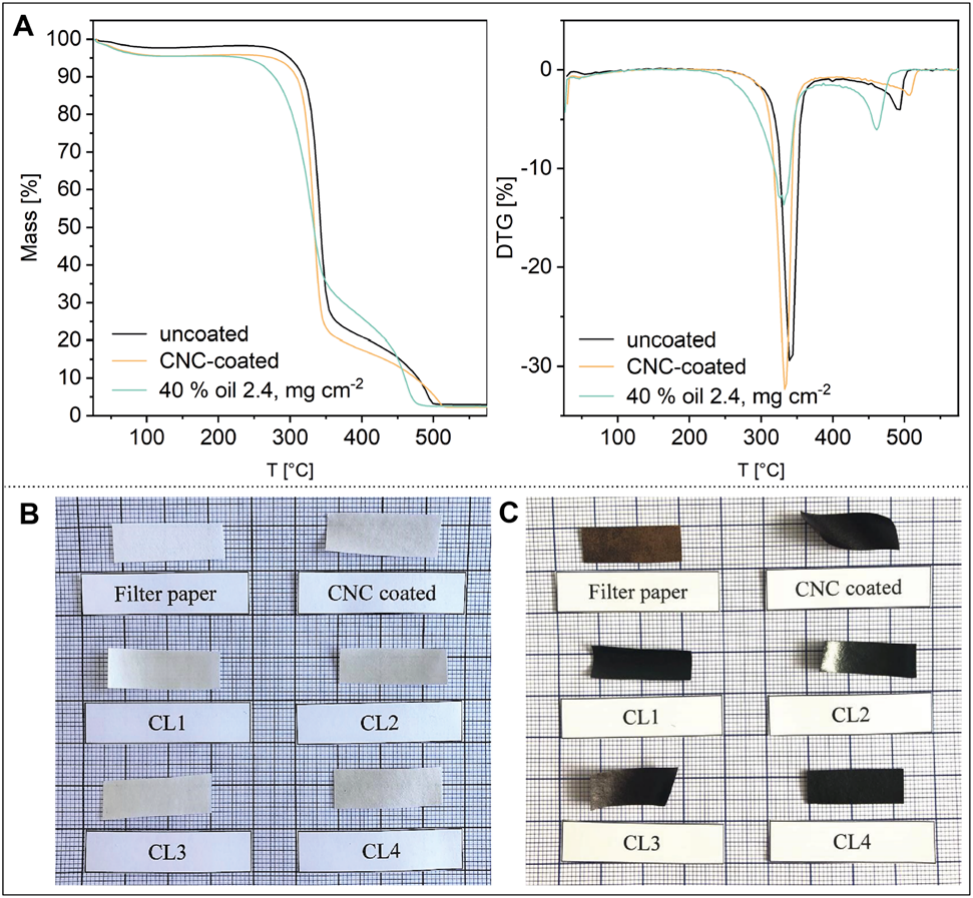
(A) Thermal analysis of uncoated, CNC-coated, and CL4-coated filter paper. (B) Uncoated and coated pieces of paper before and (C) after keeping them at 280 °C for 15 min. Thermal degradation occurs faster in the coated paper, as indicated in the dark black color. The thermal degradation of the linseed oil contained in the coating at a lower temperature than the paper causes shrinkage of the thermally degraded coating layer, resulting in rolling of the cuts.

Paper often suffers thermal stress during processing or application. To see how the emulsion coating affects the thermal degradation behavior of the paper, thermogravimetric analysis (TGA) was performed on the uncoated filter paper, a CNC-coated paper, and an emulsion-coated paper (Fig. 4).

The coating of paper with CNCs and the emulsion, here CL4 (2.4 mg cm^−2^), only slightly decreases the degradation maximum from 339 °C to approximately 332 °C in both cases. The onset of degradation, unaffected by the pure CNC coating, shows a more significant shift towards 280 °C for the emulsion-coated paper compared to 309 °C for the uncoated paper, in good agreement with the onset of thermal degradation of pure linseed oil.^21^ The inclusion of linseed oil therefore decreases flame retardancy of the paper to a small extent. As seen in Fig. 4B and C, the charring of the coating layer at lower temperatures compared to the paper also causes a loss of dimensional stability of the paper when heating it close to degradation temperature. This effect is more pronounced in the coatings with higher linseed oil contents and is due to shrinkage of the thermally degrading layer on top of the paper.

## 3 Conclusions

In this work, gas barrier properties of cellulose nanocrystals were combined with their ability to emulsify linseed oil in order to produce a hydrophobic protective paper coating. The presence of CNCs in the continuous phase increases the viscosity of the emulsion and prevents creaming, resulting in an emulsion that is stable over several months. The shear-thinning properties of CNCs also ensure a suitable rheology for blade-casting of the coating at a potential larger scale. The incorporation of a dry mass oil content of 40% to 82% produced a hydrophobic and non-porous surface with a water contact angle of up to 100°. Water vapor transmission rates showed a decreasing trend with increasing oil content at area densities higher than 0.8 mg cm^−2^ and reached values of down to 80 g m^−2^ d^−1^for the highest tested area density and oil content. We demonstrate a facile way based on bio-based and bio-degradable materials to hydrophobize paper surfaces, which could also be promising for wood treatment, with the cellulose-based emulsion promising a good adhesion to it.

## 4 Experimental

### 4.1 Preparation of CNCs

Cationic CNCs were prepared according to Jaekel *et al.*^14^ using never-dried pre-fibrillated pulp as a cellulose source. Briefly, wet bleached Kraft pulp provided by MERCER Stendal GmbH, Arneburg, Germany (64 wt% water) was mixed with a reactive eutectic medium composed of ammonium formate (Alfa Aeser) and lactic acid (90% in water, Acros Organics) in a molar ratio of 2 : 1 and reacted at 160 °C for 4 h in a stirred autoclave. The cellulosic product was precipitated and washed through cycles of redispersion in acetic acid (5 vol%) and centrifugation at 12 000*g* until a colorless supernatant was obtained. The washing was continued with ethanol in the same manner, before the solvent was exchanged to water.

### 4.2 CNC-stabilized linseed oil in water emulsions

Cold-pressed linseed oil (NaturPura, from a local supermarket) was emulsified in an aqueous CNC dispersion using an IKA T18 digital ULTRA-TURRAX^®^ high-speed lab disperser (IKA-Werke GmbH & CO. KG, Staufen, Germany) at 15 000 rpm for one minute. Paper was coated by casting different volumes of the emulsion on a filter paper (Hahnemühle, thin, medium fast, type 595, 110 mm) placed in a glass Petri dish of the same size so that no material was lost during the casting. The paper was dried at room temperature for 2 days. The coated paper was hot-pressed at 60 °C and a pressure of 3 bar between two sheets of packing paper after 7 days to allow the linseed oil to cure and avoid its absorption into the packing paper.

### 4.3 Imaging

SEM micrographs were obtained on a Gemini 1550, Zeiss AG (Oberkochen, Germany) at an accelerating voltage of 3.00 kV. For CLSM, linseed oil was stained with nile red (abcr GmbH, Karlsruhe, Germany) at a concentration of approximately 25 μg mL^−1^ before emulsification. CLSM images of the as-prepared emulsions were recorded on a Leica TCS SP8 X (Leica Microsystems, Wetzlar, Germany) using an excitation wavelength of 514 nm for Nile red and an emission detection range of 550-600 nm.

### 4.4 Water contact angle measurements

The water contact angle *θ*_w_ was measured using a Krüss contact angle measuring system G 10 (A. Krüss Optronic GmbH, Hamburg, Germany). The coated paper was fixated on a microscopic glass side with double-sided adhesive tape to ensure an flat horizontal surface. Droplets with a volume of 1 μL were placed on the surface and *θ*_w_ was evaluated by the software. Each measurement was repeated at least three times.

### 4.5 Water vapor transmission rate tests

Water vapor transmission rates (WVTR) were determined following the German industry norm DIN 53122-1 using TQC sheen permeability cups with a sample surface of 10 cm^2^ (Industrial Physics, New Castle, Delaware, US). Silica gel (230-400 mesh particle size, Sigma-Aldrich) was dried for 24 h at 80 °C before placing 5.0 g of it into the cup's reservoir. The coated paper was places coated-side-up on the cup and sealed with the screw lid. The assembly was weighed and placed into a desiccator containing a saturated solution of KCl. The resulting climate was a relative humidity of 85% at approximately 20 °C. After an equilibration time of 3 days, the cups were taken out of the weighed at intervals of 8 h to 24 h to determine the mass change Δ_m_. The results were plotted on a Δ_m_-time curve and the weighing was repeated until at least three consecutive values were on a straight line with a deviation of less than 5%.

### 4.6 Rheology

Rheological measurements were conducted on a Physica MCR-301 rheometer (Anton Paar GmbH, Graz, Austria) using a parallel plate setup (12 mm diameter). Gap spacing was 0.5 mm. Flow curves were recorded at a shear rate from 0.01 s^−1^ to 100 s^−1^.

### 4.7 Thermogravimetric analysis

TGA of uncoated and coated paper was conducted using a thermo microbalance TG 209 F1 Libra (Netzsch, Selb, Germany) in air atmosphere with a constant flow of 100 mL min^−1^. Approximately 2 mg of the dried sample was heated in an aluminum crucible from 25 to 600 °C at a heating rate of 10 °C min^−1^.

## Conflicts of interest

There are no conflicts to declare.

## Supplementary Material

RA-013-D3RA01412F-s001
