# Supplementary material for: Cationic CNC-stabilized Pickering emulsions of linseed oil for hydrophobic coatings
Source: RSC Adv. 2023 Jun 5;13(25):16860–6. doi: 10.1039/d3ra01412f (PMC10240255; doi:10.1039/d3ra01412f)
Supplement: RA-013-D3RA01412F-s001 [file RA-013-D3RA01412F-s001.pdf]

Electronic Supplementary Material (ESI) for RSC Advances.
This journal is © The Royal Society of Chemistry 2023

Supplementary Information for

Cationic CNC-stabilized Pickering emulsions of linseed oil for hydrophobic coatings

Esther E. Jaekel,*[a] and Svitlana Filonenko[a]

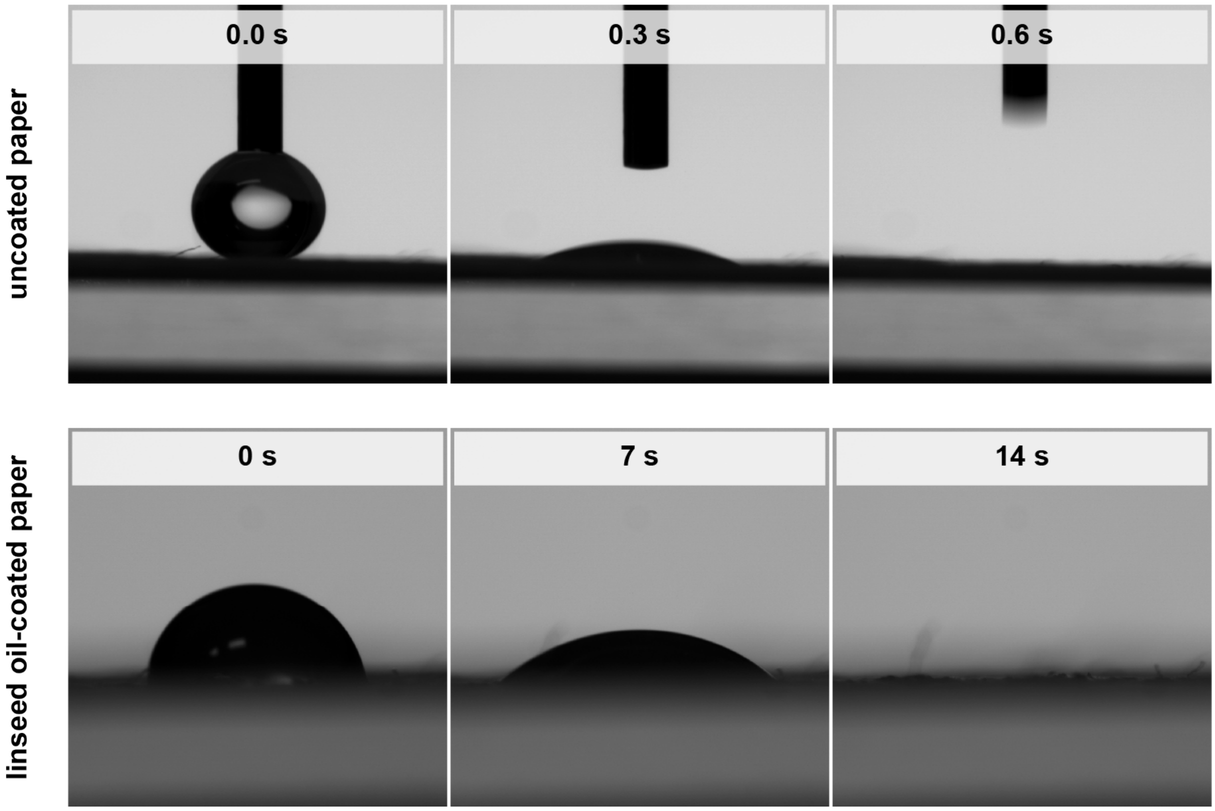

Figure S1: Photographs of sessile drop experiment on uncoated filter paper and filter paper soaked in only linseed oil.

[0a] *Am Mühlenberg 1, 14476, Potsdam, Germany. Tel: +49 331 567-9559; E-mail: esther.jaekel@mpikg.mpg.de*

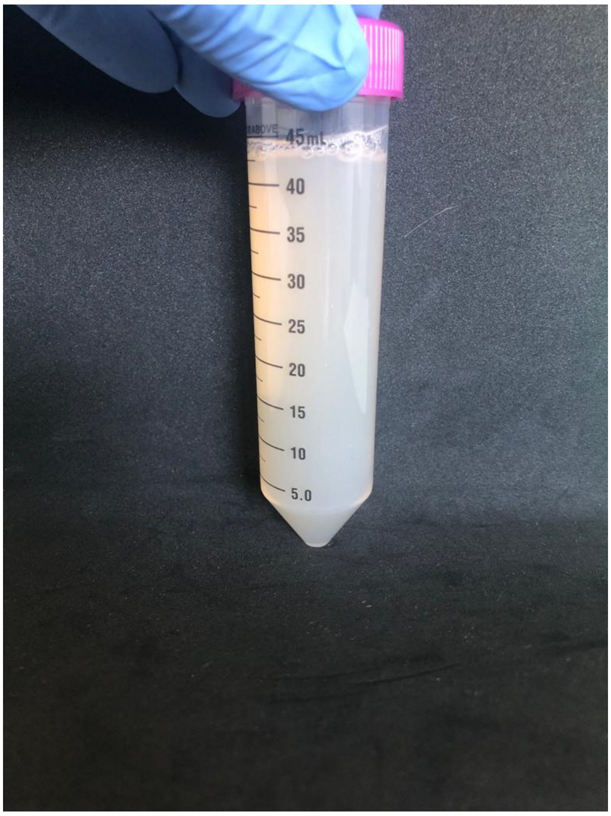

Figure S2: Photograph of CL4 after three months shows no sign of phase separation.
